# Characterisation of the tumour microenvironment and PD-L1 granularity reveals the prognostic value of cancer-associated myofibroblasts in non-invasive bladder cancer

**DOI:** 10.1080/2162402X.2024.2438291

**Published:** 2024-12-19

**Authors:** Carmen G. Cañizo, Félix Guerrero-Ramos, Mercedes Perez Escavy, Iris Lodewijk, Cristian Suárez-Cabrera, Lucía Morales, Sandra P. Nunes, Ester Munera-Maravilla, Carolina Rubio, Rebeca Sánchez, Marta Rodriguez-Izquierdo, Jaime Martínez de Villarreal, Francisco X. Real, Daniel Castellano, Cristina Martín-Arriscado, David Lora Pablos, Alfredo Rodríguez Antolín, Marta Dueñas, Jesús M. Paramio, Victor G. Martínez

**Affiliations:** a Urology Department, University Hospital ‘12 de Octubre’, Madrid, Spain; b Molecular and Translational Oncology Division, Biomedical Innovation Unit, CIEMAT, Madrid, Spain; c Centro de Investigación Biomédica en Red Cáncer (CIBERONC), Madrid, Spain; d Institute of Biomedical Research, University Hospital ‘12 de Octubre’, Madrid, Spain; e Cancer Biology and Epigenetics Group, Research Center of IPO Porto (CI-IPOP)/CI-IPOP@RISE (Health Research Network) Porto Comprehensive Cancer Center Raquel Seruca (Porto.CCC), Porto, Portugal; f Cell Technology Division, Biomedical Innovation Unit, CIEMAT, Madrid, Spain; g Centro de Investigación Biomédica en Red Enfermedades Raras (CIBERER), Madrid, Spain; h Epithelial Carcinogenesis Group, Spanish National Cancer Centre-CNIO, Madrid, Spain; i Departament de Medicina i Ciències de la Vida, Universitat Pompeu Fabra, Barcelona, Spain; j Oncology Department, University Hospital ‘12 de Octubre’, Madrid, Spain; k Scientific Support Unit, Research Institute I+12, University Hospital 12 de Octubre, Madrid, Spain

**Keywords:** Bladder cancer, cancer-associated fibroblasts, Myofibroblasts, PD-L1, tumor microenvironment

## Abstract

High-risk non-muscle-invasive bladder cancer (NMIBC) presents high recurrence and progression rates. Despite the use of Bacillus Calmette-Guérin gold-standard immunotherapy and the recent irruption of anti-PD-1/PD-L1 drugs, we are missing a comprehensive understanding of the tumor microenvironment (TME) that may help us find biomarkers associated to treatment outcome. Here, we prospectively analyzed TME composition and PD-L1 expression of tumor and non-tumoral tissue biopsies from 73 NMIBC patients and used scRNA-seq, transcriptomic cohorts and tissue micro-array to validate the prognostic value of cell types of interest. Compared to non-tumoral tissue, NMIBC presented microvascular alterations, increased cancer-associated fibroblast (CAF) and myofibroblast (myoCAF) presence, and varied immune cell distribution, such as increased macrophage infiltration. Heterogeneous PD-L1 expression was observed across subsets, with macrophages showing the highest expression levels, but cancer cells as the primary potential anti-PD-L1 binding targets. Unbiased analysis revealed that myoCAF and M2-like macrophages are specifically enriched in high-grade NMIBC tumors. The topological distribution of these two cell types changed as NMIBC progresses, as shown by immunofluorescence. Only myoCAFs were associated with higher rates of progression and recurrence in three independent cohorts (888 total patients), reaching prediction values comparable to transcriptomic classes, which we further validated using tissue micro-array. Our study provides a roadmap to establish the landscape of the NMIBC TME, highlighting myoCAFs as potential prognostic markers.

## Introduction

Bladder cancer presents an estimated number of prevalent cases of 491,243 worldwide for both sexes (https://gco.iarc.fr/en). Nonmuscle-invasive bladder cancer (NMIBC) accounts for approximately 70% of all bladder cancers. Patients diagnosed with high-risk NMIBC are treated by transurethral resection of the tumor followed by immunotherapy with Bacillus Calmette-Guérin (BCG). Despite the relatively good clinical response, these patients undergo constant invasive surveillance and repeated surgeries due to the high rates of recurrence and progression, which greatly impacts the economic cost of this diseases^1^ and quality of life of the patients.^2^ Despite many efforts to improve risk stratification of NMIBC patients,^3,4^ there is a limited ability to predict which tumors are most likely to recur and/or progress.^5^ While transcriptomic classification of primary NMIBC tumors has shown good results in terms of prognosis,^6–9^ these analysis focus mostly on cancer cells alone, leaving the tumor microenvironment (TME) less explored. Several works have studied the TME in advanced bladder cancer (BLCA),^10–14^ but this topic has been neglected in non-muscle invasive BLCA (NMIBC), specially for the nonimmune compartment of the TME. Hence, a better characterization of the TME in NMIBC is important in order to fully understand the biology of these tumors and improve their management.

The use of anti-programmed death ligand-1 (PD-L1) check point inhibitors is rising as a novel treatment in high-risk NMIBC due to limited efficacy of BCG therapy.^15^ Despite promising preliminary results, the response to these check point inhibitors varies among patients.^16,17^ While assessment of PD-L1 expression is valuable for patient stratification in certain cancers, its usefulness in BLCA is a matter of debate,^18^ especially in NMIBC.^19,20^ Besides, not only tumor cells but other TME subsets express PD-L1, which may have an impact on therapy response and prognosis.^21^

We hypothesized that a better characterization of the TME composition and PD-L1 expression in NMIBC may help understanding the tumor biology and find new prognostic biomarkers and targets. While presence of T cell subtypes is well characterized in NMIBC,^20,22^ we focused on less studied myeloid and nonimmune populations. Here, we provide a reference map of the subsets in NMIBC and quantified PD-L1 expression in all cell populations. We found that alpha-smooth muscle actin (aSMA)-expressing cancer-associated myofibroblasts (myoCAFs) serve as prognostic biomarkers in these patients.

## Materials and methods

### Ethics approval and consent to participate

This study was approved by Drug Research Ethics Committee of the University Hospital “12 de Octubre” (registry number 19/402) and performed in accordance with the Declaration of Helsinki. Informed consent was obtained from all patients.

### Patients and samples

We recruited 98 patients with BLCA diagnosis between December 2019 and December 2022 at an academic tertiary referral hospital. Patients with muscle-invasive bladder cancer (MIBC), carcinoma in situ and those samples with insufficient quality/quantity were excluded. Tumor resections were not performed en-bloc, as subsampling was necessary for the completion of pathology analysis and other parallel tests. The study was enriched for high-grade, high-risk tumors based on the greater clinical need for this population. Clinical and demographic data are summarized in supplementary table S1. Sex was not considered as a biological variable in our dataset. Nonetheless, we did generate two separate analysis for males and females when validating the results using transcriptomic data.

### Tissue digestion and antibody staining for flow cytometry

Digestion of tissue biopsies is detailed in the supplementary methods section. Zombie aqua (Biolegend) was used for viability. Immunofluorescence staining was performed by incubating the cells in PBS 1% BSA, 0.01% Sodium azide, in the presence of saturating amounts of fluorochrome-conjugated antibodies for 30 minutes at 4°C. Cyto-Fast™ Fix/Perm Buffer Set (BioLegend) was used for intracellular stainings. Antibodies and reagents used can be found in supplementary table S2. Samples were run in a Fortessa X20 flow cytometer (BD Biosciences) at CIEMAT flow cytometry facilities and analyzed using the FlowJo software (FlowJo, LLC) and OMIQ (Dotmatics). Computational analysis of flow cytometry data can be found in the supplementary methods file.

All samples were stained with the full panel minus anti-PD-L1 (FMO panel) to stablish the cutoff for positivity. The percentage of PD-L1+ was calculated according to the following formula:

*%PD-L1+ = %PD-L1full panel - %PD-L1FMO panel*

### Immunofluorescence

Antigen retrieval of 17 FFPE BLCA sections corresponding to 9 patients (9 primary tumors plus 8 recurrences/progressions) (4 TaG1; 1 TaG2; 7 T1G2; 1 T1G3; 2 T2G2; and 2 T2G3) was performed using a pressure cooker (Dako, Agilent Technologies). Primary antibodies listed in supplementary table S2 were incubated overnight. We used 4′,6-diamidino-2-phenylindole (DAPI) to stain nuclei and images were taken with a Zeiss Axioimager 2 fluorescence microscope. Cytometric analysis of 4-parameter immunofluorescences was carried out using the Qupath software^23^ as indicated in the supplementary methods section.

### Single cell RNA-seq analysis

Single cell data from Chen et al. (GSA: HRA000212) was analyzed from raw fastq data to obtain bladder cancer associated macrophage and CAF subsets.^13^ The dataset consisted of 8 primary bladder tumor tissues classified as low-grade (2) and high-grade (6) along with 3 adjacent normal mucosae samples, all obtained at the Union Hospital of Tongji Medical College, Huazhong University of Science and Technology, Wuhan, China. Fully detailed analytical methods can be found in the supplementary methods section. Gene enrichment analysis of significantly regulated genes in iCAFs and myoCAFs (supp table S3) was run using the enrichR online platform^24^ for the gene set libraries reactome 2022, molecular function 2023 and TF-Gene Co-occurrence.

### Transcriptomic data

The following three independent transcriptomic cohorts were subjected to subset association with recurrence/progression: Robertson et al,^6^ UROMOL 2021^7^ and de Jong et al.^9^ Robertson et al cohort consists of 73 T1 high grade bladder cancer samples from Northwestern Memorial Hospital, of which 13 were associated to CIS. All patients were treated with at least 6 administrations of BCG. The UROMOL project is a European multicentre prospective study of NMIBC with a total of 862 tumors (613 Ta, 238 T1, 11 carcinoma in situ). Median follow-up for patients without progression was 49 months and 10.3% progressed to MIBC. Treatment varied among patients in this cohort. De Jong et al cohort consisted of tumor samples from patients with primary high grade-NMIBC who had received at least 5/6 BCG induction instillations at four different Dutch hospitals (Erasmus University Medical Center Rotterdam, Franciscus Gasthuis and Vlietland Rotterdam, Amphia Breda, and Reinier de Graaf Gasthuis, Delft) and one Norwegian hospital (Stavanger University Hospital). This cohort includes 283 patients, of which 62 presented concomitant CIS, and were further classified as high-risk (124) and very-high risk (159).

### Tissue microarray

Cores from formalin-fix paraffin embedded tissue blocks of NMIBC were used to construct tissue microarrays (TMA; 1.5-mm core diameter), with at least two duplicate cores per case (29 patients in total), using a standard manual method (Beecher Instruments).^25^ All cores were reviewed by a pathologist to confirm the presence of representative tumor tissue. Demographic, clinical and pathological data is summarized in supplementary table S4. Antigen retrieval was performed using a pressure cooker (Dako, Agilent Technologies). Monoclonal anti-aSMA (reference in table S2) was incubated overnight. The IHC signal was amplified with a biotin-avidin-peroxidase system (ABC Elite Kit Vector) and visualized using diaminobenzidine (DAB Kit, Vector Laboratories).

### Statistical analysis

Comparisons were performed using the Wilcoxon – Mann – Whitney test (for two groups) or the Kruskal – Wallis test (for more than two groups) with Dunn´s multiple comparison test. The Spearman correlation analysis method was used to determine the correlation strength and direction between variables.

The survival analyses were performed using the Kaplan – Meier method. Differences between groups were tested using the log-rank test. A Cox proportional hazards model was fitted to estimate hazard ratio (HR) and the corresponding 95% confidence interval (CI). A multivariable model was created with all confounding and relevant factors.

All analyses were done using Stata InterCooled for Windows version 16 (StataCorp), GraphPad Prism version 9 for Windows (GraphPad Software) and R (version 4.2.1) and a level of significance of 5%.

## Results

### Clinical and pathological information

**Figure 1.**
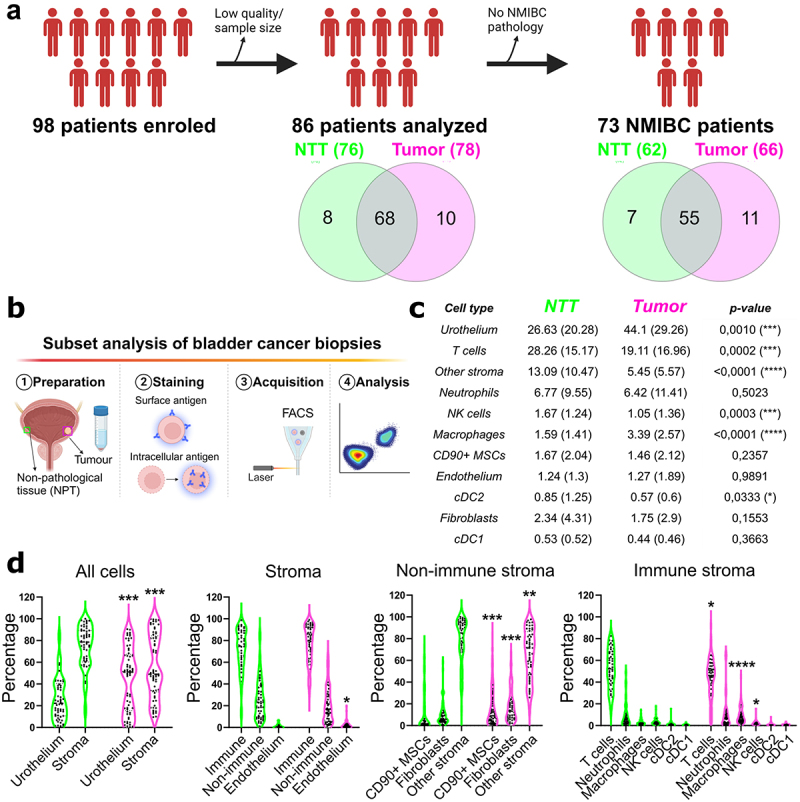
Analysis of cell subset frequencies from total and cellular compartments.

We prospectively collected and processed 98 fresh tumor and non-tumoral tissue (NTT) samples according to the schematic in Figure 1a. After diagnosis and quality control, we included 66 NMIBC tumor samples and 62 NTT biopsies in the final analysis (84.4% matched tumor-NTT ratio). Experimental pipeline for flow cytometry analysis is represented in Figure 1b. Supplementary Table S1 offers a detailed summary of clinical and histopathological information.

a) Patient enrollment and sample analysis. 98 patients were enrolled for the prospective study of which 86 patients were analyzed by flow cytometry, excluding samples from 12 patients due to low quality/availability. After pathology analysis, 73 NMIBC patients were included in the final analysis. b) Schematic representation of the prospective study design. c) Mean and standard deviation (in brackets) of the frequencies from total cells for each cell subset is shown. NTT, non-tumoral tissue. d) Frequencies of cell subsets within the corresponding cell compartment. Each dot represents one patient. NTT is colored in green, tumor in magenta. Statistical analysis in B and C was done by Wilcoxon–Mann–Whitney test. *p-value < 0.05; **p-value < 0.005; ***p-value < 0.0005; ****p-value < 0.0001. MSC = mesenchymal stromal cells; NK cells = natural killer cells; cDC = conventional dendritic cells.

### Tumour microenvironment landscape of NMIBC

We designed two multicolor panels to detect immune and nonimmune subsets, including PD-L1 expression, identifying 11 cell types. Gating strategy is in supplementary figure S1A-B. Relative proportions varied between NTT and tumors (Figure 1c). NTT samples had abundant T cells (28.26 ± 15.17%). Tumors had primarily cancer cells (44.1 ± 29.5%) and T cells (19.11 ± 16.96%). Stromal cells, T cells, NK cells, and cDC2 decreased in tumors, while macrophages increased, in accordance to literature.^26^ To mitigate urothelial cell content impact, we examined cellular compartments separately (Figure 1d). TME showed increased endothelial cells, suggesting angiogenesis. All nonimmune stroma subsets differed significantly between NTT and tumors. Immune compartment changes paralleled whole tissue results, indicating differential recruitment in tumors. We found increased fibroblast activation and M2-like differentiation of macrophages in tumors, suggesting cancer-associated inflammation in NMIBC (supplementary figure S1c).

Although muscle-invasive tumors (MIBC) are normally regarded as having a different biology and natural history, some studies point at these tumors being derived from NMIBC.^7^ To analyze NMIBC TME changes as tumors progress, we included 8 early MIBC tumors (pT2G3) in the analysis for a comparison between pT stages (supplementary figure S2a-c). Stromal infiltration increased from pTa to pT2, but leukocyte enrichment remained unchanged (supplementary figure S2a,b). Only CD90+ Mesenchymal Stromal Cells (MSCs) and Natural Killer (NK) cells significantly associated with pT stage, showing higher proportions in T2 tumors (supplementary figure S1d). Regarding tumor grade, cDC2 decreased, while CD90+ MSCs and CAFs increased in G3 tumors versus G1 tumors (supplementary figure S2d-f). Our results reveal that both invasion stage and cell grade affect bladder cancer microenvironment.

### Increased PD-L1 expression in NMIBC comes from the TME

**Figure 2.**
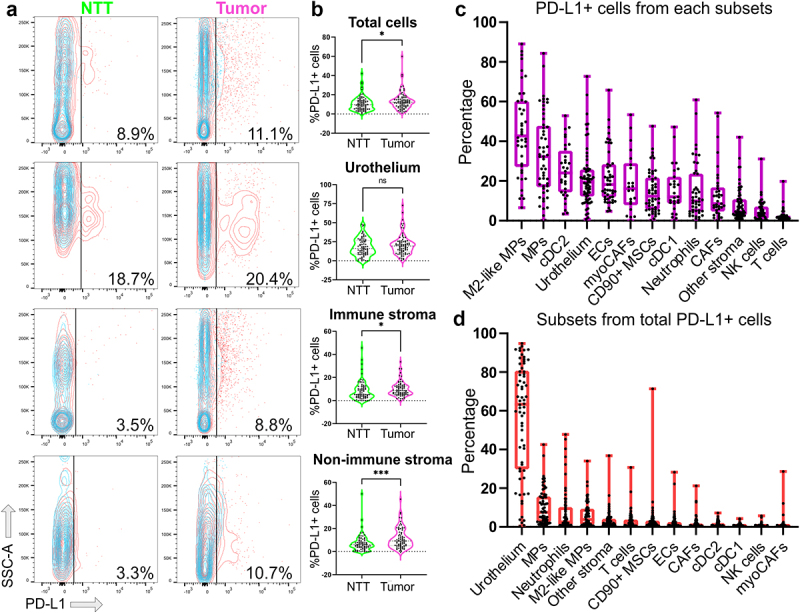
Heterogeneous expression of PD-L1 in cancer and TME cells in NMIBC.

Anti-PD-L1 therapies have emerged for NMIBC management, prompting PD-L1 expression assessment in tumor compartments. NMIBC presented elevated bulk PD-L1+ cells compared to NTT (Figure 2a,b). The increase in PD-L1+ cells in tumors stemmed from both immune and nonimmune infiltrates, as cancer cells showed similar PD-L1+ proportions to urothelium. Macrophages had the most PD-L1+ cells, with anti-inflammatory M2-like macrophages particularly rich in PD-L1 (Figure 2c). cDC2 and tumor cells followed, averaging 20% (±14.42) PD-L1+ cells, along with nonimmune stromal cells. MyoCAFs had a higher proportion of PD-L1+ cells than total CAFs. We then identified key anti-PD-L1 treatment targets in NMIBC by gating out total PD-L1+ cells, and then applying subset analysis. Cancer cells emerged as the primary binding target, constituting 60% of PD-L1+ cells (Figure 2d). Neutrophils and T cells accounted for 10% of anti-PD-L1 targets, underscoring the need to further investigate PD-L1 role in TME subsets. No significant correlations were found between cancer stage or grade and PD-L1+ cell proportions (supplementary figure S3).

a-b) Percentage of PD-L1+ cells was calculated using fluorescent minus one controls (blue overlay) for all cells and main cellular compartments, and representative examples (a) and dispersion plots (b) are shown. Each dot represents one patient data. Comparisons by Wilcoxon–Mann–Whitney test. *p-value < 0.05; ***p-value < 0.0005; ns non-significant. SSC-A =  scatter signal channel area. NTT, non-tumoral tissue. c) box plots showing percentage of PD-L1 positive cells within the indicated cell subsets. Each dot represents one patient. Median and interquartiles are shown. Subsets are ordered from higher median of PD-L1+ cells to lowest. D) PD-L1 positive cells were gated from total cells and subset analysis was run in this set of cells. Each dot represents one different patient. Median and interquartiles are shown. Subsets are ordered from higher to lower frequency median. MP = macrophages; cDC = conventional dendritic cells; ECs = endothelial cells; CAFs = cancer-associated fibroblasts; MSCs = mesenchymal stromal cells; myoCAFs = cancer-associated myofibroblasts.

### M2-like macrophages and myoCAFs are associated to high grade NMIBC

**Figure 3.**
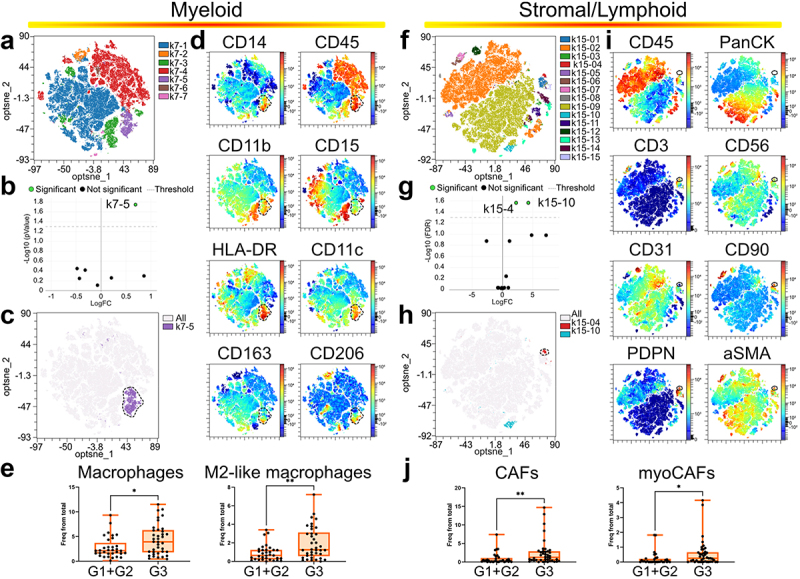
Total and specific macrophage and fibroblast subsets are enriched in high grade NMIBC. Computational analysis of flow cytometry data from the myeloid panel (a–e) and stromal/lymphoid panel (f-j). a and f) Dimensions reduction and clustering is shown. B, G) G1/G2 versus G3 comparison to determined clusters underrepresented/enriched in G3 tumors. c, h) Differentially represented cell clusters are shown in the optSNE maps. d, i) Color-coded optSNE maps show the expression of the indicated markers. Dotted lines highlight differentially represented clusters. e, j) Results from manual gating validation for the indicated cell subsets. Each dot represents one different patient. Median and interquartiles are shown. Statistical analysis by Wilcoxon – Mann – Whitney test. *p-value < 0.05; **p-value < 0.005.

We used flow cytometry data to identify markers linked to aggressive NMIBC. Comparing NTT and tumor samples, we validated the computational analysis (supplementary figure S4). Focusing on tumor samples, optimized clustering revealed multiple high-grade enriched cell clusters (Figure 3). In the myeloid panel, cluster k7–5 exhibited macrophage markers and variable M2-like markers (Figure 3a–d). Manual gating confirmed the enrichment of total and M2-like macrophages in high-grade NMIBC (Figure 3e). For the stromal/lymphoid dataset, two high-grade enriched clusters emerged, but only one could be verified with the available markers (Figure 3f–i). Cluster k15–04 showed high CD90 and podoplanin expression, indicative of cancer-associated fibroblasts (CAFs), and aSMA expression (Figure 3h,i). We performed manual gating on fibroblasts with high expression of aSMA to confirm the enrichment of total CAFs and myoCAFs in grade 3 NMIBC (Figure 3j), although a small proportions of other CAF subsets might be included in this gate. Our unbiased analysis identified two cell types with distinct phenotypes enriched in high-grade NMIBC, holding promise as potential prognostic biomarkers.

### M2-like macrophages and myoCAF topology changes with tumour progression

**Figure 4.**
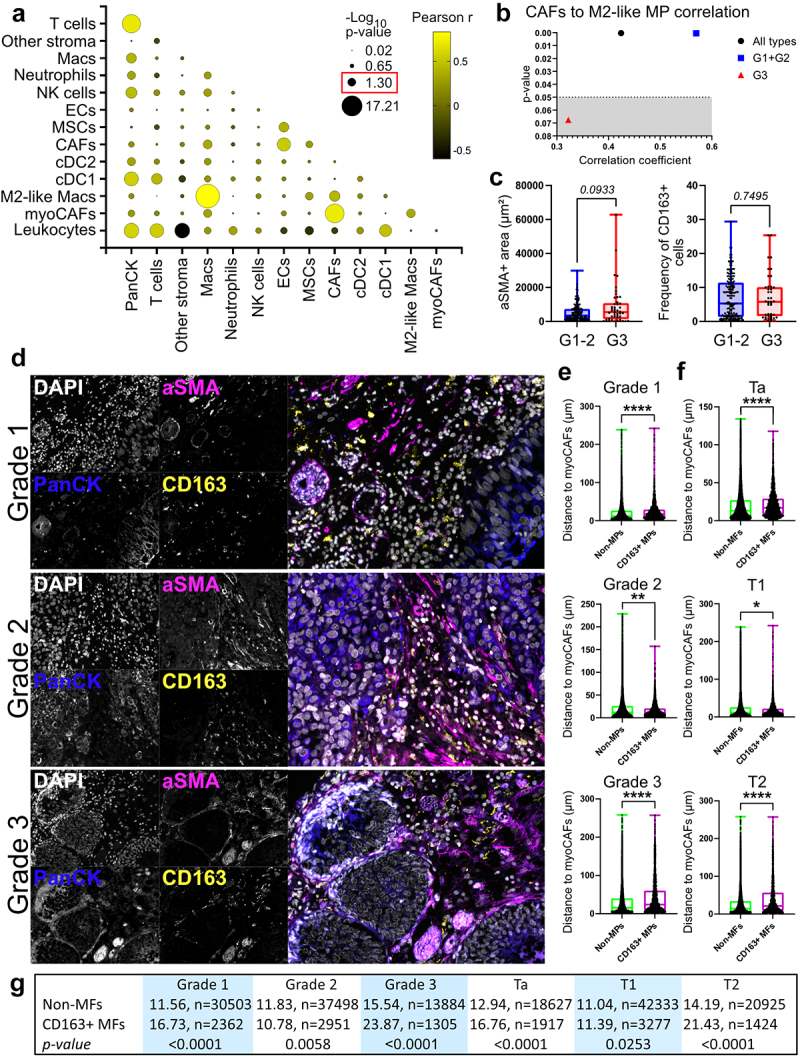
CAF-macrophage interactions decrease in high-grade NMIBC.

We did a correlation analysis on the frequency of the different cell populations detected in NMIBC. We found that the proportion of cancer cells showed high positive correlation to that of immune cells mostly and only to myoCAF within the nonimmune compartment (Figure 4a). T cells were also positively correlated to cDC1, which is in line with the interactive nature of these two cell types. We found negative association between stromal cells MSCs, endothelial cells and non-classified cells to leukocytes in general. Nonetheless, endothelial cells were associated to MSCs and CAFs. Reports show macrophage-fibroblast crosstalk in solid tumors,^27^ but it is unexplored in NMIBC. We observed a positive correlation between macrophages and CAFs in NMIBC, both total and the differentiated subtypes (Figure 4a), suggesting co-regulation of these populations. Cancer cells were the only other population that correlated myoCAFs, which may suggest specificity on the recruitment/differentiation of myoCAFs. Of note, the correlation between CAFs and M2-like macrophages decreased in high-grade tumors (Figure 4b). To investigate this, we analyzed 17 tissue sections from an independent cohort (4 TaG1; 1 TaG2; 7 T1G2; 1 T1G3; 2 T2G2; and 2 T2G3). We used aSMA and CD163 as markers for myoCAFs and M2-like macrophages, which are 60% associated to the respective subsets in the majority of patients (sup Figure 5). Similar to our flow cytometry data, we show that image quantification hinted at more myoCAFs in G3 tumors, while we could not confirm higher percentage of CD163+ macrophages (Figure 4c). Analyzing their spatial relationship, CD163+ macrophages were farther from myoCAFs in G1 tumors (Figure 4d,e). This changed drastically in G2 tumors with closer proximity, while G3 tumors presented increased separation. This may indicate co-evolution of these tow cell types in intermediate NMIBC. We found similar results with respect to pT stage (Figure 4f,g) indicating that the evolving microenvironment dynamics observed might be linked to both tumor biology and invasiveness.

(a) Bubble plot showing correlation between all cell types analysed in all NMIBC samples. Pearson r is color-coded with yellow being the highest value. Diameter of the bubble refers to the -Log10 of the p-value (1.3 equals a p-value of 0.05), with bigger bubbles correlating with lower p-value. (b) p-value against correlation coefficient of M2-like macrophage (MP) and CAF frequencies in all samples and G1–2 and G3 groups. Dotted line marks statistical significance for data points above (gray area non-significant). (c-e) 17 FFPE tissue sections (4 TaG1; 1 TaG2; 7 T1G2; 1 T1G3; 2 T2G2; and 2 T2G3) corresponding to 9 patients (9 primary tumors plus 8 recurrences/progressions) were stained for cytokeratins (PanCK), M2-like macrophages (CD163) and myoCAFs (aSMA), plus DAPI. (c) the area of aSMA staining, excluding the vasculature, and percentage of CD163 positive cells is shown. (d) Representative examples for grade 1, 2 and 3 tumor stainings. (e) quantification of the minimum distance to non-vasculature aSMA+ areas for all CD163-negative (non-MPs) and CD163-positive (CD163+ MPs) cells. P-values and significance (asterisks) by Wilcoxon–Mann–Whitney test are shown. **p-value < 0.005; ****p-value < 0.0001.

**Figure 5.**
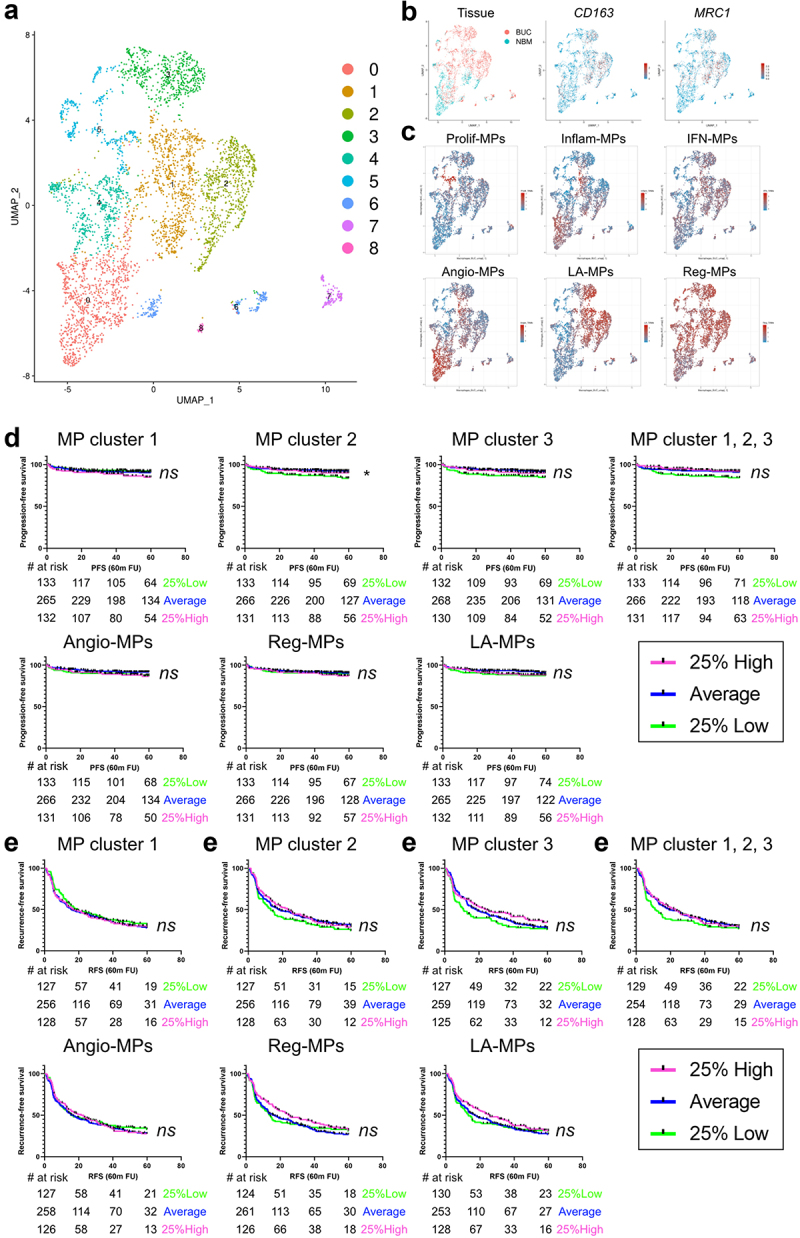
Tumor-associated macrophage subsets do not correlate to prognosis in NMIBC.

Single cell data from Chen et al. was analyzed to extract macrophages (MP) as an independent object. Functional analysis was performed using VISION R package and the resultant signature scores were incorporated as metadata to the seurat object. UMAP plots color-coded for macrophage clusters (a), bladder urothelial carcinoma (BUC) versus bladder normal mucosa (BNM) and CD163 and MRC1 expression (b), gene-signature scores for described tumor-associated macrophage subsets. d-e) Kaplan–Meier plots showing probability of recurrence-free and progression free survival of patients stratified according to gen-signature scores for the indicated macrophage subsets. Log-rank (Mantel-Cox) test was used for statistical significance. *p-value < 0.05; ns, not significant. Angio-mp, angiogenic macrophages; LA-MP, lipid associated macrophages; reg-mp, regulatory macrophages; prolif-mp, proliferative macrophages; inflam-mp, inflammatory macrophages; IFN-MP, interferon macrophages. FU, follow-up.

### Macrophage subset gene signatures fail to predict NMIBC prognosis

Next we tested the prediction value of macrophages by exploring transcriptomic data from NMIBC patients. First, we used single-cell RNA-seq data^13^ to identify macrophage clusters. Clusters 1, 2, and 3 showed M2 marker expression and high scores for immunosuppressive macrophage subsets,^28^ thus resembling those identified in our flow cytometry data (Figure 5a-c). We challenged one of the largest NMIBC transcriptomic cohorts, the UROMOL cohort (530 NMIBC patients)^7^ with gene signatures for all relevant macrophage subsets (supplementary table S3) and most comparisons resulted in no association to recurrence/progression-free survival (Figure 5d,e). We only found that patients performed worse when showing low scores for macrophage cluster 2. Overall, our results suggest that immune suppressive macrophage subsets are unlikely to be a significant prognostic factor in NMIBC.

### Transcriptomic analysis of CAFs shows ECM related features in bladder cancer

**Figure 6.**
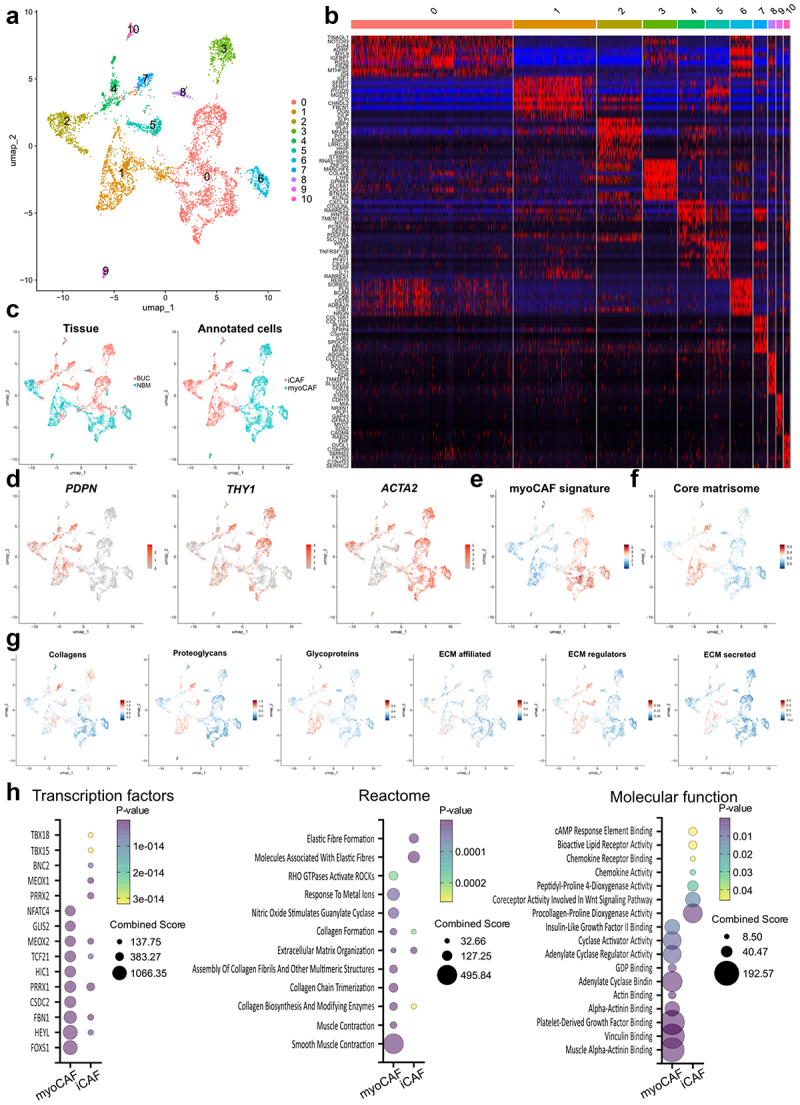
CAF subset analysis in BLCA reveal ECM and contractility features in myoCafs.

Next, we further explored the role of myoCAFs in BLCA by analyzing publicly available scRNAseq data.^13^ Cells classified as fibroblasts (positive for *COL1A1* expression) were sub grouped into 11 clusters, finding a number of differentially regulated genes in each group (Figure 6b). The eleven clusters were divided between the two main CAF subsets, i.e. iCAFs and myoCAFs, as previously annotated (Figure 6a,c), with clusters 0, 1 and 2 presenting a mixture of normal and tumoral tissue, therefore containing normal fibroblasts. We show that among the cellular markers employed in flow cytometry for the identification of CAFs and myoCAFs, *ACTA2* (aSMA) was strongly associated to myoCAFs in this cohort (Figure 6d). We next generated a myoCAF gene signature based on genes with a differential expression of 2 fold-change or more, that was widely represented in myoCAF subgroups (Figure 6e). Since modulation of the extracellular matrix (ECM) is one of the main features of CAFs, we interrogated this scRNAseq dataset for the expression of matrisome genes.^29^ We found that core matrisome genes and all matrisome categories were better represented in iCAFs as compared to myoCAFs, while Collagen genes were more evenly distributed (Figure 6f,g). Gene enrichment analysis for transcription factor co-regulation revealed that both iCAFs and myoCAFs share a similar dependence on master regulators of fibroblast development and phenoconversion, such as TCF21, MEOX2 and PRRX1. The two CAF subsets show active Notch signaling as evidenced by enrichment on HEYL-regulated genes, which has been linked to myofibroblast differentiation^30,31^ (Figure 6h). NFATC4, MEOX2 and PRRX1 also regulate process associated to myofibroblasts such as ECM stiffness, TGF-β signaling, angiogenesis, and Wnt pathway,^32–35^ while PRRX2 appears to be strongly involved in Wnt5a signaling, which correlates with the relevance of this pathway in BLCA iCAFs.^11^ Similarly, TBX18 is associated to development and activation states in fibroblasts,^36^ while BNC2 seems to negatively regulate matrix deposition.^37^ Finally, HIC1 and FOXS1 hubs are exclusively enriched in myoCAF and have been shown as early development markers in fibroblasts, also linked to contractility of these cells,^38,39^ and direct regulation of aSMA and myofibroblasts differentiation.^40^ In this line, reactome and molecular function enrichment analysis further support active contractility in myoCAFs, together with ECM organization and adherence, while iCAFs display a phenotype also associated to chemotaxis and Wnt signaling (Figure 6h). Altogether, myoCAFs in bladder cancer are strongly associated to contractility and ECM remodeling, as has been observed in other tumors.

Single cell data from Chen et al. Was analyzed to extract macrophages COL1A1-expressing fibroblasts as an independent object. Functional analysis was performed using VISION R package and the resultant signature scores were incorporated as metadata to the seurat object. UMAP plots color-coded for CAF clusters (a). Top-10 differentially expressed genes for each cluster are shown in a heatmap (b). UMAP plots color-coded for bladder urothelial carcinoma (BUC) versus bladder normal mucosa (BNM) and iCAF/myoCAF classification (c), plus *PDPN*, *THY1* and *ACTA2* expression (d), gene-signature scores for the generated myoCAF signature (e) and described matrisome categories (f-g). h) Bubble charts show results for the enrichment analysis for the indicated libraries using genes with significant increased expression in myoCAFs and iCAFs as input.

### MyoCAFs serve as cellular predictors of bad prognosis in NMIBC

**Figure 7.**
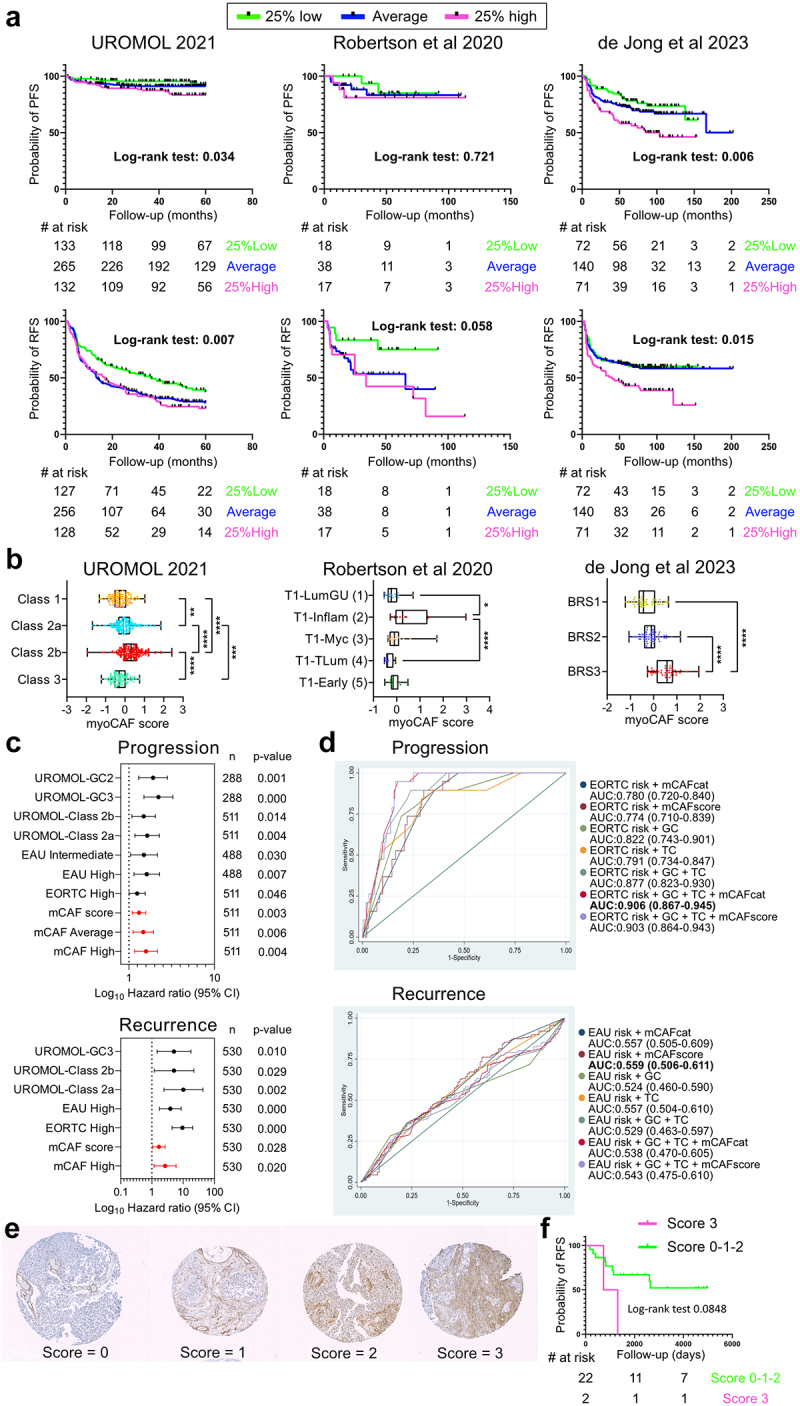
Abundance of myoCAFs associates with bad prognosis in NMIBC.

We assessed patient cohorts with gene signatures for total CAFs (panCAFs), inflammatory CAFs (iCAFs), and aSMA-expressing CAFs (myoCAFs) in bladder cancer.^11,13^ PanCAFs and iCAFs showed no prognostic value for recurrence-free (RFS) and progression-free survival (PFS) (supplementary figure S6a-b). Conversely, the myoCAF signature was strongly associated with worse PFS and RFS (Figure 7a), confirmed using an alternative myoCAF signature in the same and independent cohort (supplementary figure S6C), and in two additional independent cohorts^6,9^ (Figure 7a). In the three cohorts analyzed here, transcriptomic classification was associated to differential prognosis. Classes 2b, and specially 2a, correlated with higher probability of recurrence and progression in the UROMOL study.^7^ While T1-Early/T1-Myc in Gordon et al,^6^ and BRS3 in de Jong et all^9^ cohorts were associated to higher recurrence and progression respectively. Therefore, we wanted to see if myoCAFs might be associated to these classification systems. We found that high myoCAF abundance correlated with aggressive transcriptomic classes in UROMOL and de Jong cohorts, and to the T1-Inflamed in Gordon et al (Figure 7b). While panCAFs and iCAFs also associated with aggressive classes in the UROMOL cohort, only myoCAFs were enriched in genomic classes linked to worse RFS and PFS (supplementary figure S6d-e). This suggests a specific link between tumor biology and TME composition related to myoCAFs. MyoCAF scores were similar in both sexes and provided equal prognostic value (supplementary figure S7). Univariate Cox regression showed myoCAF category and score as strong predictors of PFS and RFS in NMIBC, with hazard ratios comparable to established predictors (Figure 7c). ROC analysis for progression prediction demonstrated that adding the myoCAF category improved the prediction power, achieving outstanding discrimination (AUC: 0.906 ± 0,037) (Figure 7d). However, we could not improve the otherwise poor prediction model for RFS. aSMA staining in a tissue micro-array of NMIBC indicated a trend toward lower RFS in patients with high myoCAF staining (Log-rank test p-value = 0.0848) (Figure 7e,f), although not statistically significant due to low sample size. These findings support that myoCAFs are associated with aggressive NMIBC and serve as potential biomarkers for recurrence and progression.

(a) a gene signature score was generated for myoCAFs to challenge two independent transcriptomic data cohorts of NMIBC. Patients were ranked according to this score and three groups were formed. Kaplan–Meier plots for probability of progression-free survival (PFS, upper panel) and recurrence-free survival (RFS, lower panels) for the three independent cohorts are shown. Log-rank (mantel-cox) test was used to calculate statistical significance between curves. (b) dotplots showing myoCAF scores for patients grouped according to cohort-specific transcriptomic classes. Each dot represents one patient. *p*-values **< 0.005; ***< 0.0005; ****< 0.0001. Statistical analysis was done with Kruskal-Wallis test with Dunn´s correction for multiple comparisons. (d-e) overview of hazard ratios calculated from univariate cox regressions of progression-free and recurrence-free survival using clinical and molecular features. Horizontal lines show 95% confidence intervals (CI). EORTC = european organisation for research and treatment of cancer, EAU = european association of urology. (d) performance of a multivariable analysis to predict progression (upper panel) and recurrence (lower panel) in the UROMOL 2021 cohort. For each curve, AUC (area under the curve) and confidence interval (in brackets) is shown. Bold letters highlight the model with the highest AUC. FU, follow-up; myoCAF-s, myoCAF score; myoCAF-c, myoCAF category; myoCAF-h, myoCAF category high; class 2a/C2a, transcriptomic class 2a. f) Representative aSMA immunohistochemistry in primary tumor sections from NMIBC patients. All sections were scored from 0 to 3 according to aSMA staining density in non-vasculature areas, to exclude pericytes. G) Kaplan–Meier plot for recurrence-free survival in patients stratified by aSMA staining score. Statistical analysis by log-rank (Mantel-Cox) test.

## Discussion

Several studies have aimed at characterizing the TME of NMIBC, often using transcriptomic-based methods.^6–8,41,42^ These analyses are informative but hard to compare across studies and are not well-suited for clinical practice. Studies using immunohistological staining are limited by the number of markers analyzed, failing to provide a comprehensive picture of the NMIBC TME landscape. Our study is the first to offer a reference map of relative proportions of 11 cell types. Comparison with non-tumoral tissue revealed cancer-associated changes in the bladder mucosae. The compartment analysis provides a clear picture of previously obscured changes in NMIBC affecting the microvasculature and increased fibroblasts and MSCs within the nonimmune stroma. The differences in the leukocyte compartment align with previous publications, showing fewer T cells but more macrophages and NK cells in tumors, which is further exacerbated in MIBC.^42,43^ This suggests a tumor-promoting role for these cells, as indicated in therapy response studies.^44–46^

Immune checkpoint inhibitors targeting the PD-1/PD-L1 pathway have shown benefits in MIBC and are promising for BCG-unresponsive high-risk NMIBC patients. They are also under investigation in BCG-naïve high-risk NMIBC.^47^ Despite good therapeutic results, using these targets as prognostic and predictive biomarkers is controversial. Most studies measure PD-L1 expression in whole tissue sections or separate only tumor and immune-infiltrating cells. Our results reveal broad heterogeneity of PD-L1 expression among TME subsets, indicating that most studies lack the necessary granularity to assess its prognostic value properly. Notably, unlike other cancers, PD-L1 expression in NMIBC cells is similar to healthy urothelium, suggesting that immune evasion via this molecule is a late event in BLCA progression.

Most studies addressing CAFs in BLCA have focused on MIBC or mixed cohorts, largely ignoring NMIBC CAFs. Our results show that fibroblasts populate NMIBC, especially in high-grade tumors, with no differences between Ta and T1. This indicates that fibroblast recruitment depends on cancer cell biology rather than the level of invasion. We found that myoCAFs, but not other CAF subsets, associate with poor prognosis in NMIBC patients, consistent with findings in MIBC.^48,49^ Mezheyeuski et al. tested CAF-associated markers in BLCA, finding associations with high-grade NMIBC and identifying fibroblast activation protein (FAP) as a predictor of poor outcome, although not when separating Ta, T1, and T2–4.^50^ Interestingly, aSMA was statistically associated with worse prognosis only for T1 tumors, aligning with our findings. Additionally, a different CAF subset, irCAFs, has been described in a mixed cohort of MIBC and NMIBC patients, associating with worse overall survival and poor neoadjuvant and immunotherapy response, likely through promoting cancer stemness.^11^ Future studies should use more discriminatory panels and resolve the spatial distribution of cell types to better characterize CAF subsets in NMIBC.

Regarding the role of myoCAF in BLCA and its association to worse prognosis, our functional characterization of this subset via analysis of scRNAseq data shows that myoCAF´s function is strongly linked to collagen assembly and contractility. This suggests that myoCAF may be regulating matrix stiffness in BLCA. Indeed, matrix stiffness has been observed before the onset and invasion of the tumor in rat bladders^51^ and higher collagen deposition and stiffness correlates with high grade in BLCA.^52,53^ Of note, matrix stiffness induces BLCA cell proliferation and epithelial-to-mesenchymal transition, which has been attributed to CAFs in an 3D *in vitro* model.^54^ Although further functional characterization needs to be done, our study and current literature indicate that myoCAFs promotes BLCA progression via modulation of the ECM.

We also found that CAF-macrophage spatial relationship changes over NMIBC-MIBC progression. According to our data, myoCAF-macrophage closeness is augmented between early NMIBC and MIBC onset, which may be indicative of parallel recruitment/differentiation of these two cell types in intermediate NMIBC. We found similar results grouping the tumors by grade 1-2-3 terminology or the clinically more relevant pT classification. While limited by the use of only one marker to identify these cell types, our results are in accordance to previous studies. Other authors have shown co-attraction of both subsets by BLCA cells via CXCL1^55^ and feedback loops via CCL2 and GM-CSF production by CAFs,^56^ which may explain our results. More recently, nicotinamide N-methyltransferase (NNMT) positive fibroblasts have been shown to mediate recruitment and differentiation of suppressive macrophages in human and mouse BLCA.^57^ Of note, cancer cells and macrophages stablish metabolic circuitries that may dictate the co-evolution of both cell types in later stages,^58^ therefore detached from CAFs, as it is the case for serine metabolism in BLCA.^59^ These observations reflect the dynamic nature of the TME as BLCA progresses, likely influenced by cancer cell intrinsic characteristics since it appears tied to tumor progression.

In conclusion, the NMIBC TME is composed by a heterogeneous distribution of cancer, immune and nonimmune cells. Our results strongly suggest that myoCAFs play an important role in NMIBC. Future studies should elucidate how and why myoCAFs associate with worse prognosis in order to find appropriate targets to modulate their function.

## Supplementary Material

Supplemental Material

## Data Availability

Raw data were generated at Centro de Investigaciones Energéticas, Medioambientales y Tecnológicas (CIEMAT). Derived data supporting the findings of this study are available from the corresponding author VGM on request.
