# Supplementary material for: Characterisation of the tumour microenvironment and PD-L1 granularity reveals the prognostic value of cancer-associated myofibroblasts in non-invasive bladder cancer
Source: Oncoimmunology. 2024 Dec 19;14(1):2438291. doi: 10.1080/2162402X.2024.2438291 (PMC11660370; doi:10.1080/2162402X.2024.2438291)
Supplement: Supplemental Material [file KONI_A_2438291_SM7455.zip › New folder/Gomez C et al Oncoimmunol Supplementary material final REVISED.docx]

**Characterisation of the tumour microenvironment and PD-L1 granularity reveals the prognostic value of cancer-associated myofibroblasts in non-invasive bladder cancer**

Carmen G. Cañizo^1^, Félix Guerrero-Ramos^1^, Mercedes Perez Escavy^2, 3, 4^, Iris Lodewijk^2, 3, 4^, Cristian Suárez-Cabrera^2, 3, 4^, Lucía Morales^2, 3, 4^, Sandra P Nunes^2, 4, 5^, Ester Munera-Maravilla^2, 3, 4^, Carolina Rubio^2, 3, 4^, Rebeca Sánchez^6, 7^, Marta Rodriguez-Izquierdo^1^, Jaime Martínez de Villarreal^3, 8^, Francisco X. Real^3, 8, 9^, Daniel Castellano^10^, Cristina Martín-Arriscado^11^, David Lora Pablos^11^, Alfredo Rodríguez Antolín^1^, Marta Dueñas^2, 3, 4^, Jesús M. Paramio^2, 3, 4 ⴕ^, Victor G. Martínez^2, 3, 4^ *

**SUPPLEMENTARY MATERIAL**

**Supplementary methods**

**Tissue digestion and antibody staining for flow cytometry**

Tissue biopsies were carefully cut into small pieces and digested using collagenase P at 200 μg/ml (Sigma-Aldrich), dispase II 800 μg/ml (thermofisher scientific) and DNase I 100 μg/ml (Sigma-Aldrich) in DMEM at 37°C in a water bath. After 30 min samples were mixed by pipetting up and down and half of the digestion media replenished by fresh until all tissue was digested. Media replacement was repeated two more times with 15 min incubations in between. After erythrocyte lysis, cell suspensions were centrifuged, resuspended in FACS buffer (PBS 2% BSA) and filtered through a 40 μm cell strainer (Corning).

**Computational analysis of flow cytometry data**

An optimal compensation matrix was applied to each data set. For automated analysis, data were automatically cleaned-up via FlowAI algorithm. Further cleaning was performed by manual gating to remove doublets, debris, and dead cells. Scaling of the data was done by arcsinh transformation (cofactor 150). Approximately 140,000 target count cells were analysed for each panel, all files equally subsampled. optSNE and FlowSOM were run using default settings, including all markers but zombie aqua and PD-L1. The optimal number of metaclusters was selected according to results from manual gating. In brief, we used the results from NTT vs tumour comparisons using manual gating as our ground truth. This allowed as to better assess efficient number of metaclusters. EdgeR algorithm was used for all two-group comparisons, using the p-value as the cut-off for statistical significance.

**Single cell RNA-seq analysis**

Reads were pseudoaligned to GRCh38 cDNA sequence assembly using ‘kallisto bus’ command (Kallisto software, default parameters) and sparse matrices were generated bustools program and BUSspaRse R package as described ^25^. Individual sample matrices loaded with Seurat package were merged in a common object. Data was normalized (sctransform and log normalization for the top 2000 variable genes) and stored as two different slots of the same Seurat object. Linear dimensionality reduction was performed using the first 20 principal components (PC) according to elbow plot visualization. Non-linear dimensional reduction (UMAP) and clustering were performed using these PCs. Cluster stability was visualized in a clustree analysis (resolution set to 0.2). FindAllMarkers was used for cell annotation. Macrophages and fibroblasts were extracted and reanalysed as an independent object. Functional analysis was performed using VISION R package ^26^ and the resulting signature scores were incorporated as metadata to the Seurat object. Construction of gene-signature scores was done using 2 and 4-fold change differentially expressed genes for each subset or by previously published gene sets, all shown in supplementary table 3.

**Cytometry analysis of immunofluorescent tissue sections**

Nineteen FFPE BLCA sections (7 G1, 9 G2 and 2 G3) were stained for aSMA, CD163, PanCK and DAPI. Cytometric analysis of 4-parameter immunofluorescences was carried out using the Qupath software. To calculate non-vasculature associated aSMA staining area, a pixel classifier was trained with 20+ tissue sections using as annotations aSMA-negative, aSMA-positive and aSMA-vasculature. Cell detection was used for deconvolution of cells using DAPI staining, and positive cell detection was run to classify CD163-positive and CD163-negative cells. The function distance to annotation 2D was applied to calculate the minimum distance of CD163-negative and CD163-positive cells to non-vasculature aSMA-positive structures.

**Supplementary figures and tables**


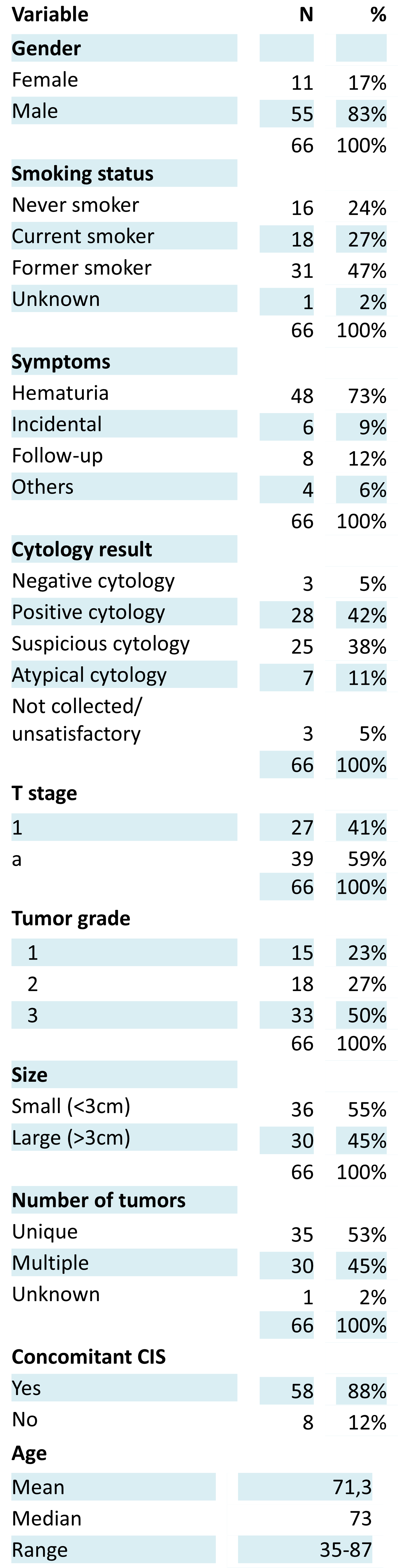


**Supplementary table 1**. Demographic, clinical and pathological data from patients included in the study regarding tumor samples only.


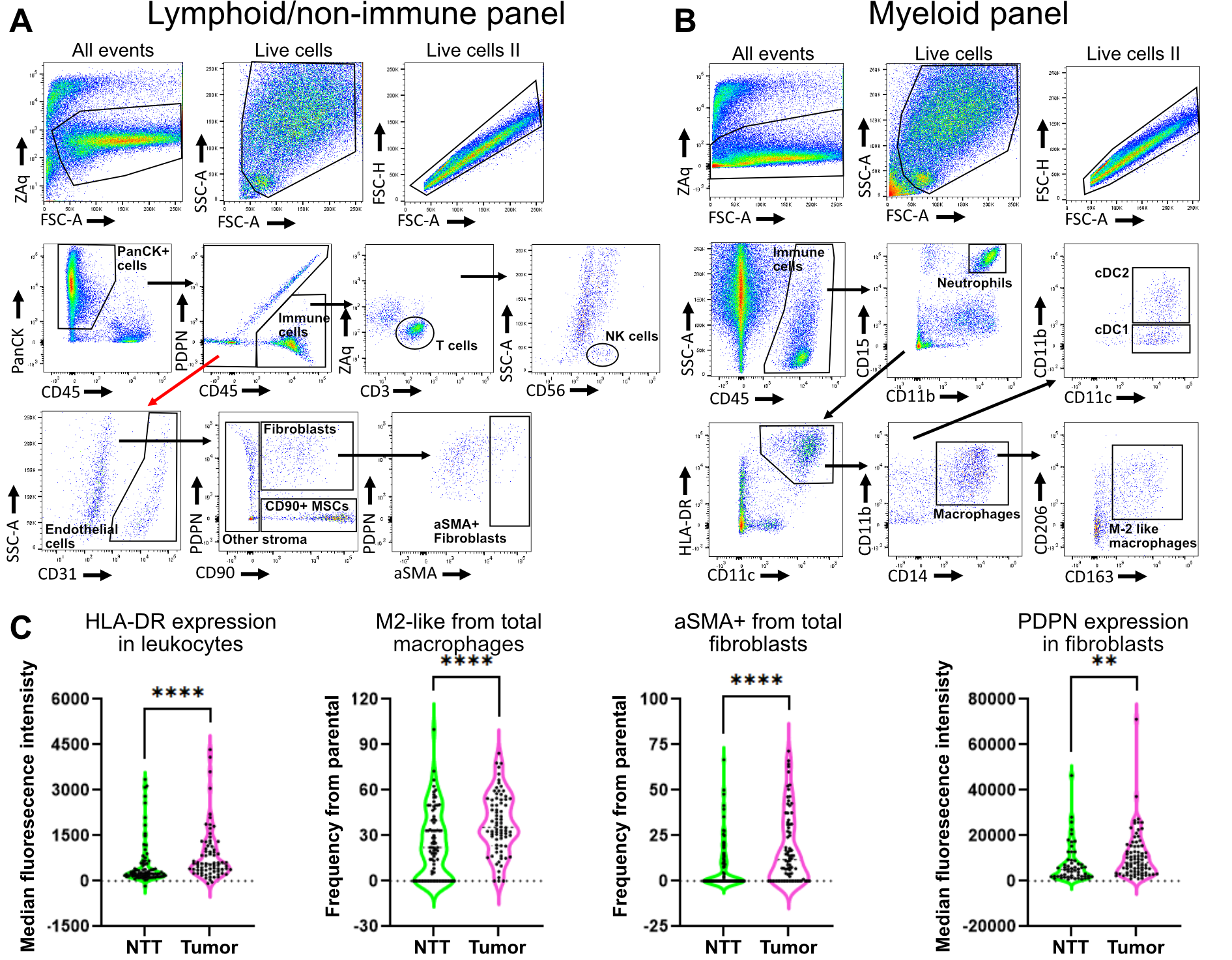


**Supplementary figure 1. NMIBC presents inflammation-associated features**. Conventional gating strategy for the quantification of lymphoid/non-immune (A) and myeloid cells (B) using two separate flow cytometry panels. C) Comparisons between non-tumoral tissue (NTT) and tumours for several inflammation surrogate markers. Frequencies from parental gates and median fluorescence intensities are shown. P-values * < 0.05; *** < 0.0005 by Wilcoxon–Mann–Whitney test. HLA-DR, Human Leukocyte Antigen – DR Isotype; PDPN, podoplanin; aSMA, alpha-smooth muscle actin; MSC, mesenchymal stromal cells.


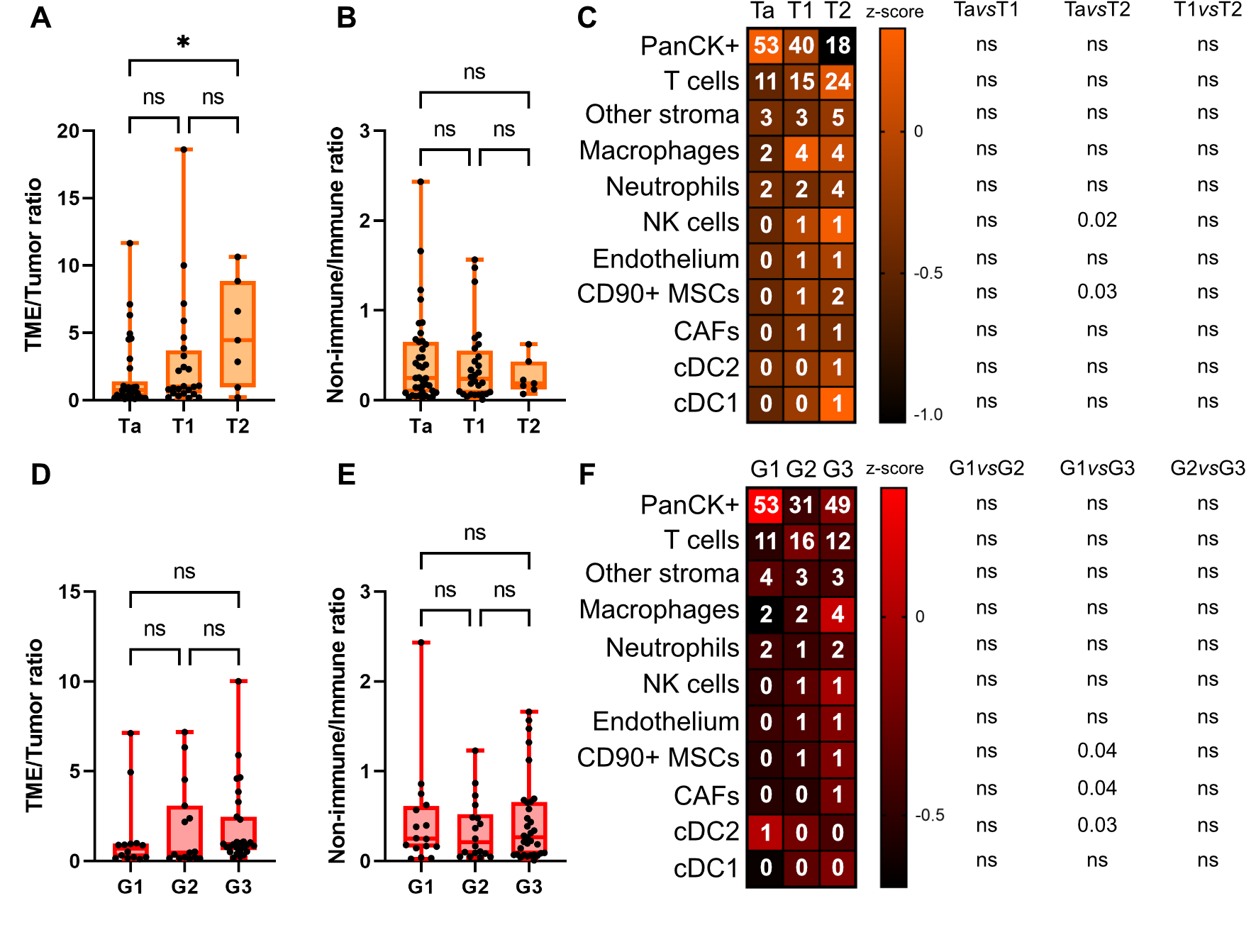


**Supplementary figure 2. Dynamics of tumour microenvironment composition along bladder cancer development.** Flow cytometry data was used to analyse frequency of cell compartments and subtypes in patients stratified by pT stage and grade. For comparisons between pT stages, 6 to 8 T2 tumours were added to the cohort. A, D) Ratios for stroma/cancer cell proportions. B, E) Ratios of non-immune/immune cell proportions were calculated from PanCK-negative cells. C, F) Heatmaps show z-score (color-coded) and medians (numbers in white) for the proportion from total cells of the indicated cell subtypes along pT stage and grade. P-values are shown for statistically significant comparisons by Kruskal-Wallis test (ns, non-significant) applying Dunn´s test for multiple comparisons.


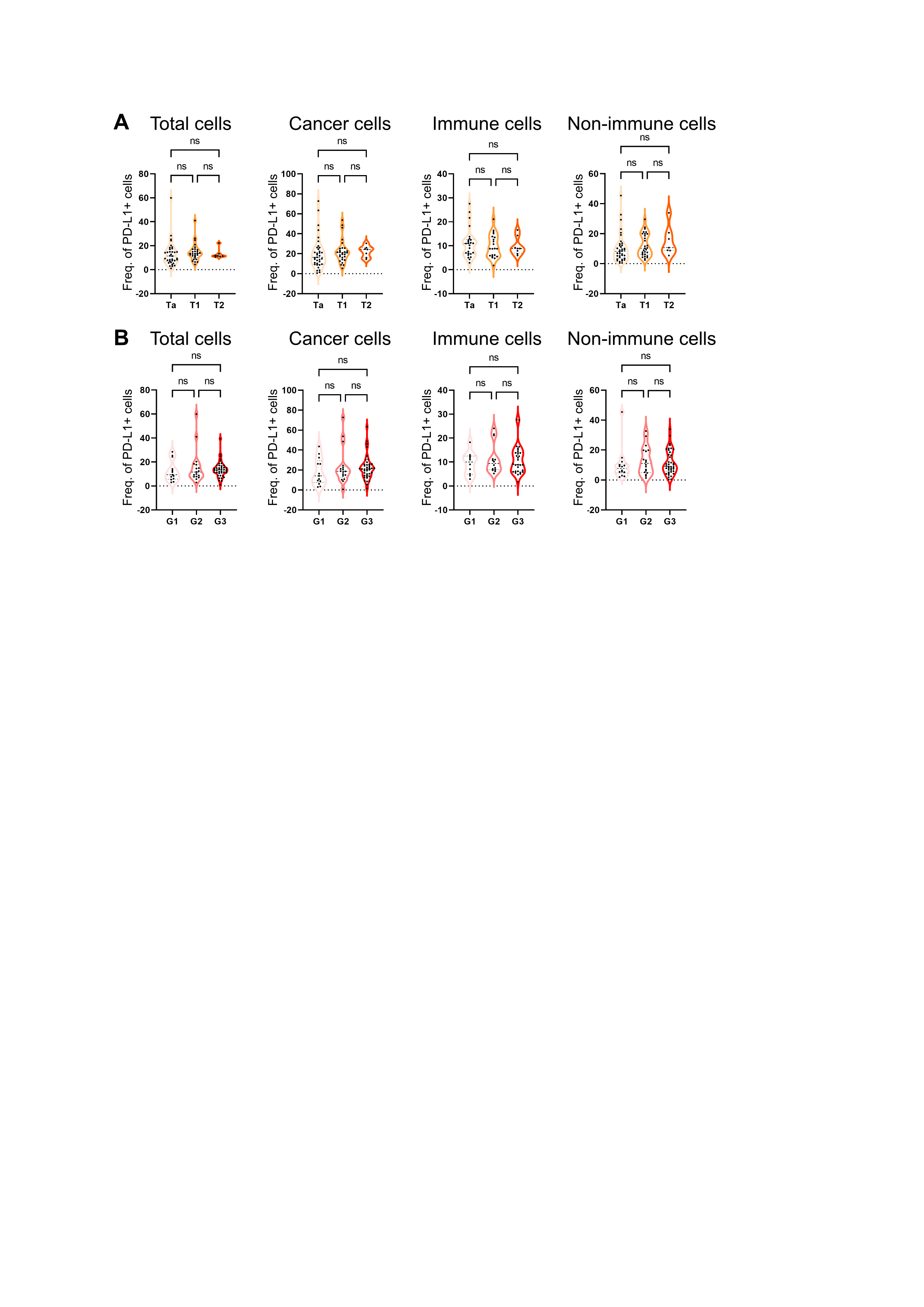


**Supplementary figure 3. No association between PD-L1 expression in different cell compartments and pT stage and grade.** Flow cytometry data was used to calculate the percentage of PD-L1+ cells within the indicated cellular compartments in patients grouped according to pT stage (A) and grade (B). Each dot represents one patient. For pT stage analysis, 6-8 T2 tumours were included. ns, non-significant by Kruskal-Wallis test.


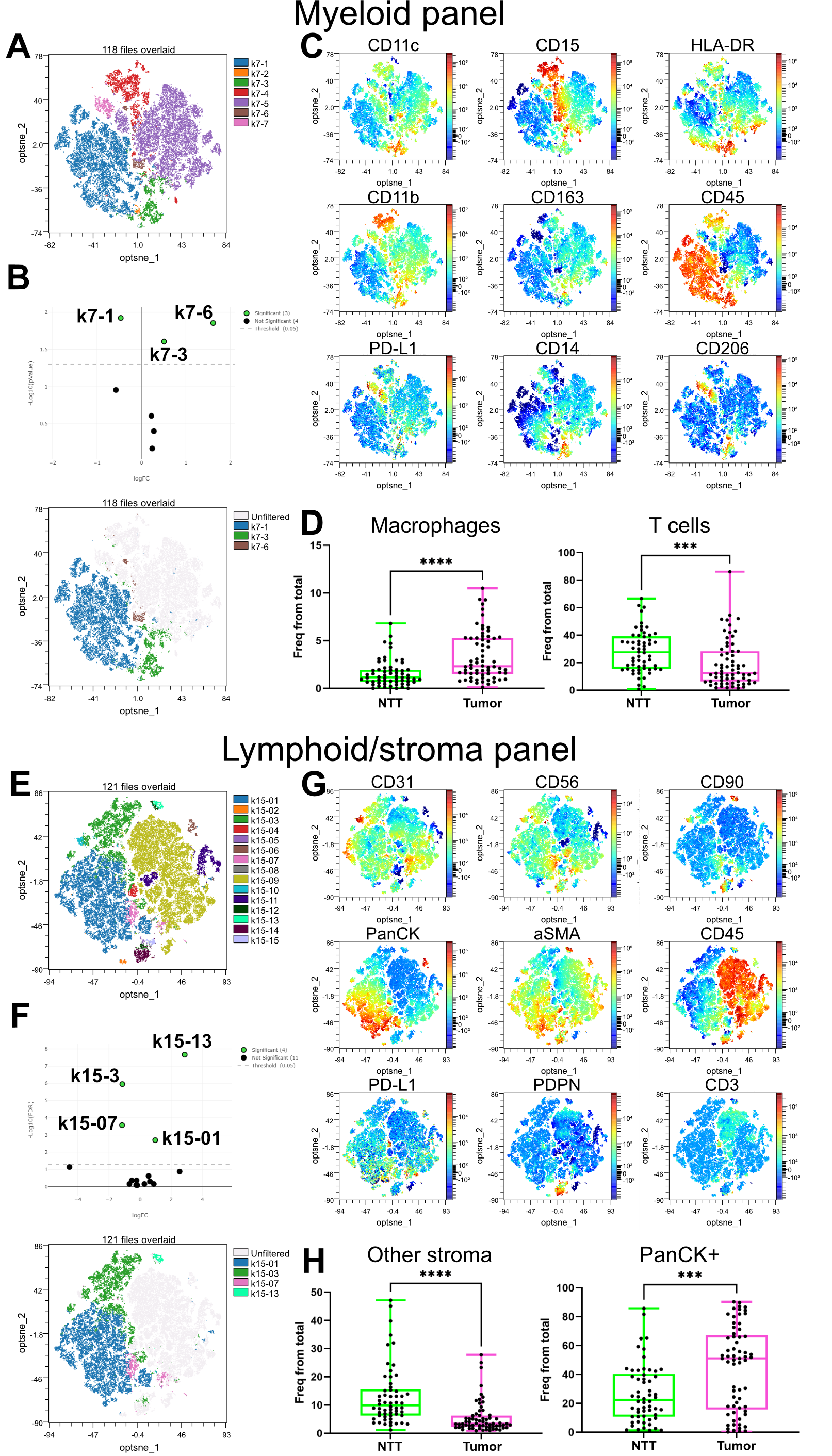


**Supplementary figure 4. Semi-supervised computational analysis of two flow cytometry dataset to compare non-tumoral tissue (NTT) and tumours.** Datasets corresponding to the flow cytometry panels for myeloid (A-C) a lymphoid/non-immune stroma cells were subjected to computational analysis using the OMIQ platform. First, reduction of dimensions was performed by optSNE and semi-supervised clustering by FlowSOM, setting the optimal number of clusters as 7 and 15 respectively. A, E) Visualisation of cell clusters overlaid in optSNE maps. B, F) Volcano plots show clusters presenting statistically different frequency in tumours by EdgeR algorithm. optSNE maps highlight over (left side) and under (right side) represented clusters in tumours. C, G) Color-coded optSNE maps showing the expression of the indicated cell markers. D, H) Validation of computational results using the manual gating strategy. D) k7-3 and k7-1 corresponds to macrophages and T cells respectively. H) k15-01 and k15-03 corresponding to cancer cells and other stroma cell subsets respectively. P-values *** < 0.0005 and **** < 0.0001 by Wilcoxon–Mann–Whitney test.


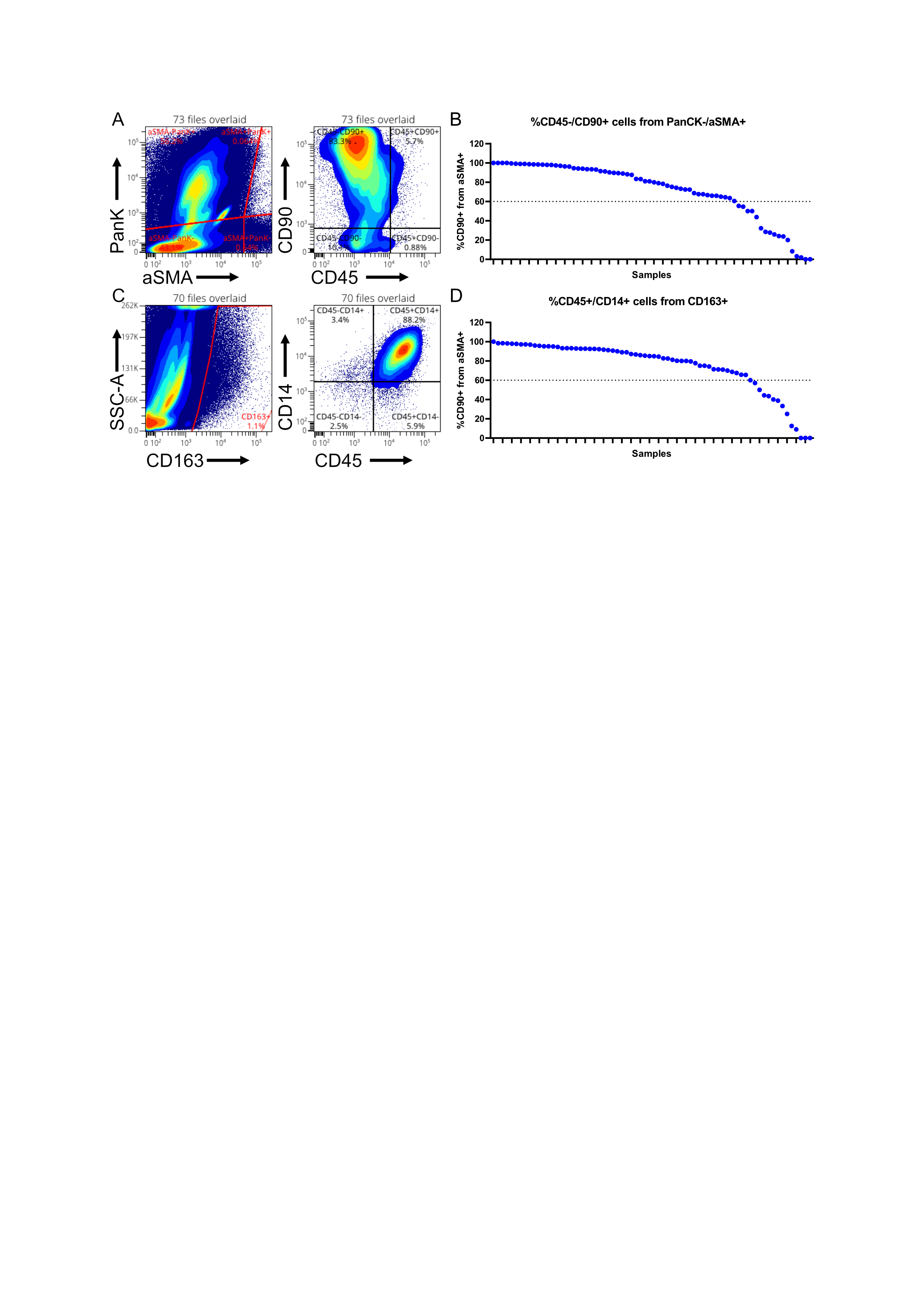


**Supplementary figure 5. Association of single markers to cellular subsets.** Datasets corresponding to the flow cytometry panels for myeloid (A-C) a lymphoid/non-immune stroma cells (C-D) were used to interrogate the exclusivity of single cell markers. A) Selection of Cytokeratin (PanCK)-negative, aSMA-positive population from total cells (left panel) and expression of CD45 and CD90 in these cells. All samples are shown concated in a single dot-plot. B) Percentage of CD45-negative/CD90-positive stromal cells within the PanCK-negative/aSMA-positive population. Each dot represent one patient. C) Selection of CD163-positive population from total cells (left panel) and expression of CD45 and CD14 in these cells. All samples are shown concated in a single dot-plot. B) Percentage of CD45-positive/CD14-positive macrophages within the CD163-positive population. Each dot represents one patient. PanK, cytokeratins; SSC-A, side scatter channel area.


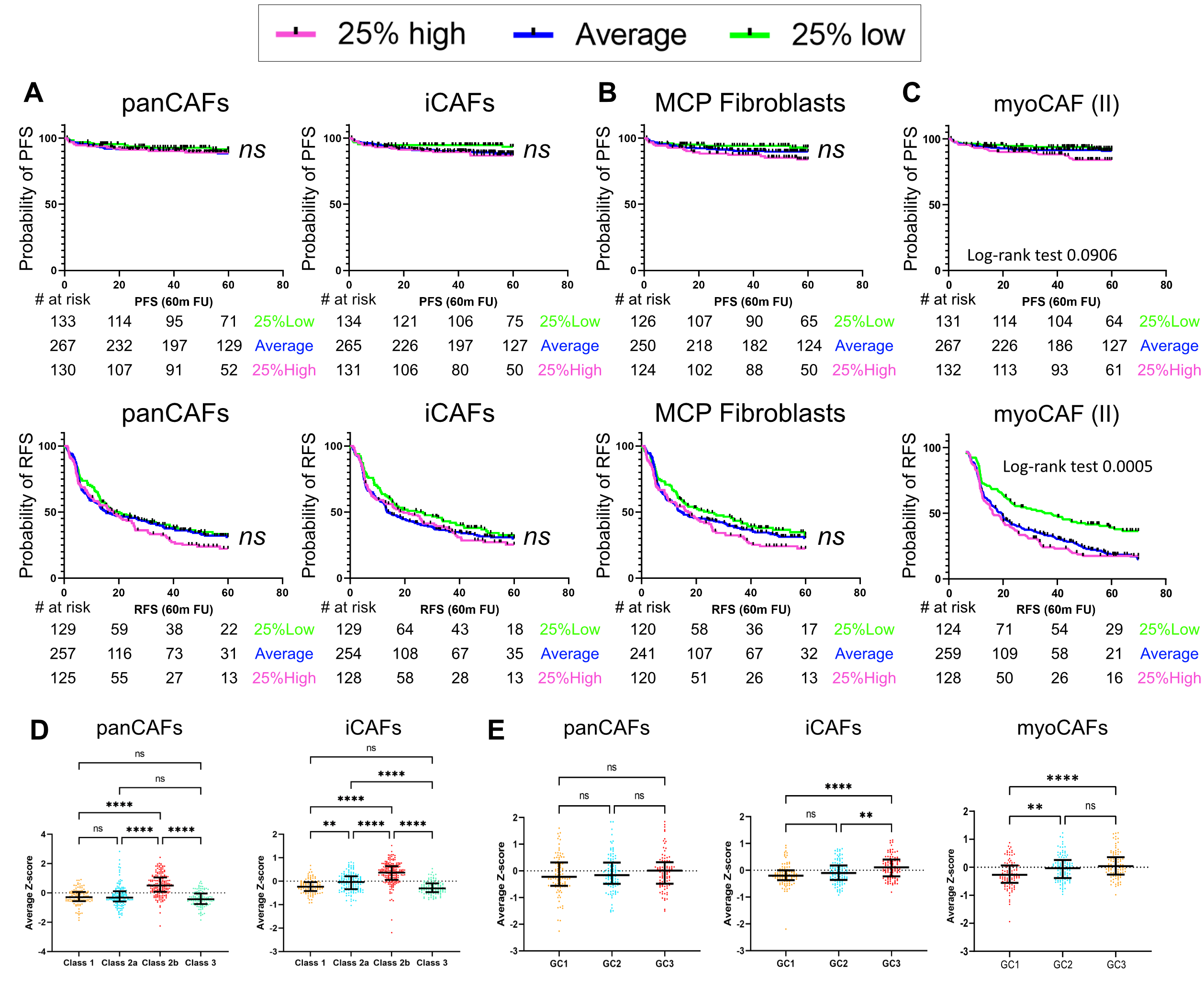


**Supplementary figure 6. Correlation of fibroblast subset gene signatures to prognosis in NMIBC.** Different gene signature scores were generated for CAF subsets to challenge clinical outcome of the UROMOL 2021 cohort of NMIBC tumours. Patients were ranked according to these scores and three groups were formed. A-C) Kaplan–Meier plots for probability of progression-free survival (PFS, upper panel) and recurrence-free survival (RFS, lower panels) for the indicated subsets are shown. Log-rank (Mantel-Cox) test was used to calculate statistical significance between curves. A) Total CAFs (panCAF) and inflammatory CAFs (iCAFs). B) MCP counter fibroblast signature. C) MyoCAF signature newly generated from Chen et al. D-E) panCAF and iCAFs gene-signature scores in patients stratified by UROMOL 2021 transcriptomic classes (D) and genomic classes (E). F) Overview of hazard ratios calculated from univariate Cox regressions of recurrence-free (upper panel) and progression-free (lower panel) survival using clinical and molecular features. Dots indicate hazard ratios and horizontal lines show 95% confidence intervals (CI). P-values and sample sizes, n, used to derive statistics are indicated. CIS, carcinoma in situ; myoCAF, cancer-associated myofibroblasts.


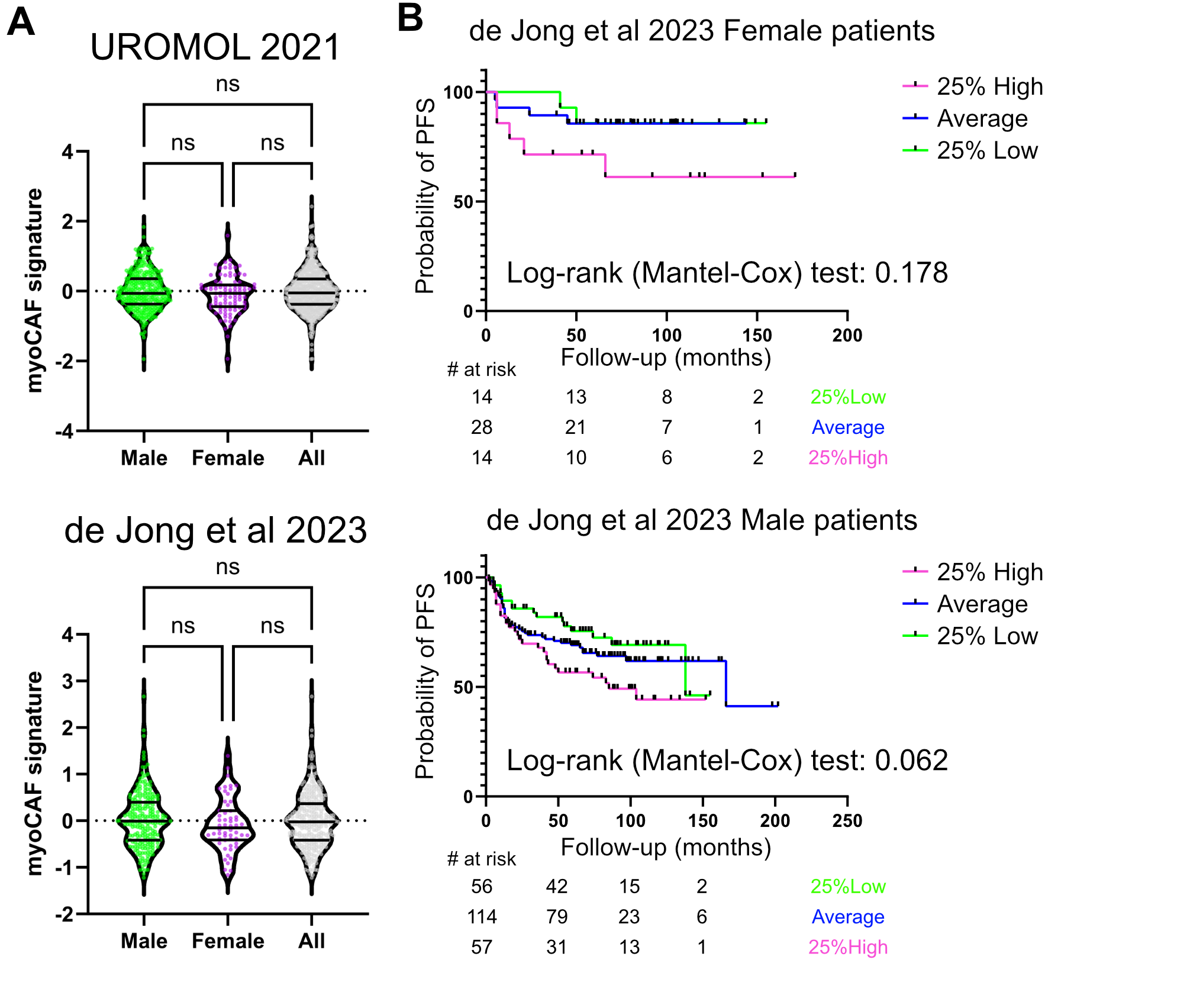


**Supplementary figure 7. myoCAF score shows similar association with poor prognosis in male and female NMIBC patients**. Gene signature scores for myoCAFs were compared between all, male and female patients in the two indicated NMIBC transcriptomic cohorts. Statistical analysis was done with Kruskal-Wallis test with Dunn´s correction for multiple comparisons. ns means non stastistical results. B) Female and male patients were ranked according to myoCAF scores and three groups were formed. Figure shows Kaplan–Meier plots for probability of progression-free survival (PFS) for the indicated subsets. Log-rank (Mantel-Cox) test was used to calculate statistical significance between curves.
